# Bibliography

**Published:** 1902-10-15

**Authors:** 


### BIBLIOGRAPHY.

The S. S. White Dental Manufacturing Co. has just issued a very complete catalogue entitled "Porcelain Teeth." It is more than a catalogue of teeth for sale, it contains an historical sketch of the manufacture of teeth, and much information, which if carefully studied by dentists would make the selection and proper adaptation of artificial teeth a more intelligent and satisfactory process. It is really a valuable work and every dentist who does this work should have a copy and study it.

THE *American Dental Journal*, published by Frink & Young, of Chicago, and edited by Dr. J. B. DICUS, is the newest dental journal. It comes out in a red, white and blue cover, which might be pretty if the printer had done his work well. The first number is labeled Volume I, Number 1, but if there is anything to indicate the date of publication we fear the blue ink must have got the best of it. It's new all through, and compared with our old fashioned notions it has some things which we could criticise, but we won't, for we know the enterprise and intelligence back of it will put them right in due time. We cordially welcome the new journal and wish it success.

"MEDICAL BOOK NEWS." P. Blackistone Son & Co., of Philadelphia, have published the first number of a bi-monthly journal intended to print information in regard to medical literature. It will contain, descriptions, reviews, list of new works, and everything of interest in this direction. A good scheme if well carried out.

